# An update comprehensive review on the status of COVID-19: vaccines, drugs, variants and neurological symptoms

**DOI:** 10.3906/biy-2106-23

**Published:** 2021-08-30

**Authors:** Ebru ÖZKAN OKTAY, Salih TUNCAY, Tuğba KAMAN, Ömer Faruk KARASAKAL, Öznur Özge ÖZCAN, Tuğçe SOYLAMIŞ, Mesut KARAHAN, Muhsin KONUK

**Affiliations:** 1 Laboratory Technology Program, Vocational School of Health Services, Üsküdar University, İstanbul Turkey; 2 Food Technology Program, Vocational School of Health Services, Üsküdar University, İstanbul Turkey; 3 Medical and Aromatic Plants Program, Vocational School of Health Services, Üsküdar University, İstanbul Turkey; 4 Medical Laboratory Techniques Program, Vocational School of Health Services, Üsküdar University, İstanbul Turkey; 5 Physiotherapy Program, Vocational School of Health Services, Üsküdar University, İstanbul Turkey; 6 Vocational School of Health Services, Üsküdar University, İstanbul Turkey; 7 Department of Molecular Biology and Genetics, Faculty of Engineering and Natural Sciences, Üsküdar University, İstanbul Turkey

**Keywords:** COVID-19, variants, neurological symptoms, drugs, vaccines, neutralizing antibodies, SARS-CoV-2

## Abstract

Various recently reported mutant variants, candidate and urgently approved current vaccines against severe acute respiratory syndrome coronavirus 2 (SARS-CoV-2), many current situations with severe neurological damage and symptoms as well as respiratory tract disorders have begun to be reported. In particular, drug, vaccine, and neutralizing monoclonal antibodies (mAbs) have been developed and are currently being evaluated in clinical trials. Here, we review lessons learned from the use of novel mutant variants of the COVID-19 virus, immunization, new drug solutions, and antibody therapies for infections. Next, we focus on the B 1.1.7, B 1.351, P.1, and B.1.617 lineages or variants of concern that have been reported worldwide, the new manifestations of neurological manifestations, the current therapeutic drug targets for its treatment, vaccine candidates and their efficacy, implantation of convalescent plasma, and neutralization of mAbs. We review specific clinical questions, including many emerging neurological effects and respiratory tract injuries, as well as new potential biomarkers, new studies in addition to known therapeutics, and chronic diseases of vaccines that have received immediate approval. To answer these questions, further understanding of the burden kinetics of COVID-19 and its correlation with neurological clinical outcomes, endogenous antibody responses to vaccines, pharmacokinetics of neutralizing mAbs, and action against emerging viral mutant variants is needed.

## 1. Introduction

Recent infectious viral diseases such as severe acute respiratory syndrome (SARS) and Ebola disease have given rise to a major threat to public health, causing significant global pandemics. Previously known severe acute respiratory syndrome (SARS) coronavirus (SARS-CoV), Middle East respiratory syndrome (MERS) coronavirus (MERS-CoV), and finally coronavirus disease 2019 (COVID-19) coronavirus SARS-CoV-2 caused severe respiratory discomfort in the last 20 years. SARS-CoV originated in the Guangdong province of China in 2002. This virus spread over five continents, infected 8098 people and caused 774 deaths. MERS-CoV emerged in the Arabian Peninsula. Then, it was detected in 27 countries and effected 2494 people with 858 deaths in (He et al., 2020; Khan et al., 2020; Walls et al., 2020). In December 2019, the novel coronavirus was firstly reported in Wuhan, Hubei province of China, and globally spread. The World Health Organization (WHO) has named the causative virus SARS-CoV-2/2019-nCoV and the related infected disease as coronavirus disease 2019 (COVID-19). Although first hopes against the COVID-19 pandemic are borne out by urgently approved vaccines, many mutant variants from global phylogenies have been reported. Moreover, neurological diseases and symptoms have been reported from COVID-19 patients, so there is a risk that the pandemic may reach an uncontrollable level. For instance, E484K mutant virus is estimated to have a 6-fold reduction in sensitivity to immune sera and an 11-fold reduction in susceptibility to convalescent sera from persons vaccinated with Pfizer/BioNTech mRNA vaccine (Collier et al., 2021).

In particular, the most updated issue we discuss here is to focus on considering how emerging mutant strains of concern will impact vaccine, drug, and monoclonal therapeutic targets in the long term to contain the pandemic.

## 2. Variants of concern (VOC)

**Figure 1:**
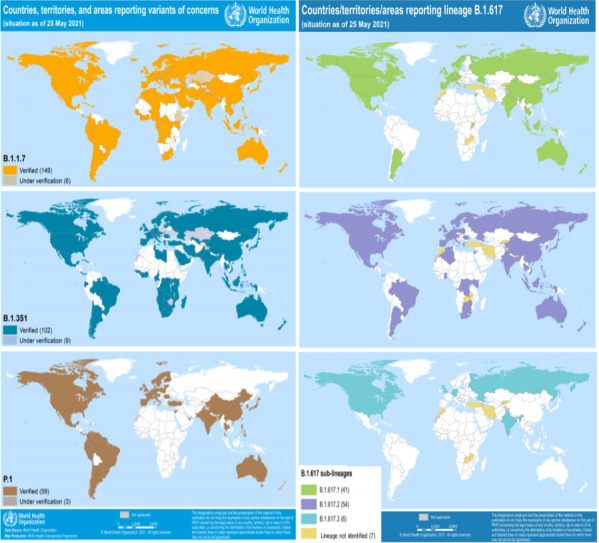
Maps of variants of concern reports as of 25 May (World Health Organization, 2021b).

Viruses generally have one of the highest mutation rates of all organisms (Almubaid and Al-Mubaid, 2021). It is known that the rate of spontaneous mutation occurrence varies between viruses. The mutation rate in RNA viruses is higher than in DNA viruses and higher in single-stranded viruses than double-stranded ones. In addition, the large genome size is correlated to the lower mutation rate (Sanjuán and Domingo-Calap, 2016). It is known that coronaviruses mutate rapidly, these mutations occur especially in the spike protein (S protein) genes. These mutations might allow the virus to evade the host’s immune response and adapt to the host environment. Alterations in the S protein may cause the formation of many variants with different virulence characteristics, as well (Saha et al., 2020). A variant that has evidence for association with increased virulence and transmissibility, decreased efficacy of available diagnostics, therapeutics, and vaccines is denominated as a variant of concern (VOC) (World Health Organization, 2021a). There have been numbers of SARS-CoV2 variants identified and reported worldwide recently. Among them, B 1.1.7, B 1.351, P.1, and B.1.617 lineages or VOCs were detected and identified in late 2020. The B 1.1.7, B 1.351, and P.1 lineages were reported in 149, 102, and 59 countries, respectively as of May 25, 2021. In the same report, the sublineages of B.1.617, B.1.617.1, B.1.617.2, and B.1.617.3 were reported in 41, 54, and 6 countries, respectively (Figure 1) (World Health Organization, 2021b). Davies et al. (2021) reported that B.1.1.7 variant was estimated to have a 43%–90% higher reproduction number than previous variants (Davies et al., 2021). B.1.351 variant was forecasted to be 1.50 (95% CI: 1.20–2.13) times more transmissible than earlier available variants (Pearson et al., 2021). P.1 variant was estimated to be 2.5 times (Coutinho et al., 2021) and 1.7–2.4 times (Faria et al., 2021) more transmissible than wild type variant. B.1.617.2 is evaluated to have at least equivalent transmissibility to B.1.1.7 according to available data for the present (Public Health England, 2021). Table summarizes some of the main characteristics of those lineages. 

**Table:** Main characteristics of VOCs (as of 25 May 2021) (World Health Organization, 2021b).

PANGO lineage	B.1.1.7	B.1.351	B.1.1.28.1 (P.1)	B.1.617*
Nextstrain clade	20I/501Y.V1	20H/501Y.V2	20J/501Y.V3	-
GISAID clade	GR/501Y.V1	GH/501Y.V2	GR/501Y.V3	G/452R.V3
Other names	VOC-202012/01	VOC-202012/02	VOC-202101/02	-
Location of first detection	United Kingdom	South Africa	Brazil / Japan	India
Date of first detection	20 September 2020	Early August 2020	December 2020	October 2020
Characteristics spike mutations	69/70del, 144del, N501Y, A570D, D614G, P681H,T716I, S982A, D1118H	D80A, D215G, 241/243del, K417N, E484K, N501Y,D614G, A701V	L18F, T20N, P26S, D138Y, R190S, K417T, E484K, N501Y, D614G H655Y, T1027I, V1176F	L452R, D614G, P681R, ± (E484Q, Q107H, T19R,del157/158, T478K, D950N)

*B.1.617 viruses are separated into three lineages (B.1.617.1, B.1.617.2, and B.1.617.3).

S protein is responsible for mediation of viral entry to host cell. Firstly, S protein binds to human ACE2 (angiotensin-converting enzyme 2) receptor via its receptor-binding domain (RBD). Then, it enzymatically becomes efficient by human proteases (Hoffmann et al., 2020a; Shang et al., 2020). Because of this role, S protein became the main target of COVID-19 vaccines. Notable spike protein mutations of VOCs are also listed in Table. This section of the manuscript focuses on especially some spike protein modifications due to its gene mutations within VOCs below.

### 2.1. Main characteristics of B.1.1.7 lineage

B.1.1.7 lineage, variant of concern 202012/01 is a variant that emerged in the UK in September 2020 and rapidly spread throughout the world. This lineage contains 23 mutations (14 nonsynonymous mutations, 6 synonymous mutations, and 3 deletions) in its genome of which eight mutations occur in S protein (Public Health England, 2020). Some of these mutations may regulate protein’s function such as N501Y, P681H, and H69/V70 deletion (Goncalves Cabecinhas et al., 2021). N501Y mutation found within the RBD in S protein of B.1.1.7 lineage may increase binding to human ACE2 (Luan et al., 2021). N501Y is estimated to increase infectivity by 52% (Zhao et al., 2021). N501Y is found in B.1.351 and P.1 lineages, as well. P681H mutation is found contiguous to the furin cleavage site at the S1/S2 in the S protein. Although the function of P681H is unknown, the cleavage of S1/S2 site is required for S protein-mediated cell-cell fusion and entry of the virus into human lung cells (Hoffmann et al., 2020b; Gómez et al., 2021). Double deletion at positions 69-70 (H69/V70 deletion) in B.1.1.7 variant was associated to affect the performance of diagnostic PCR assays using the spike glycoprotein (S) gene as a target.^1^https://www.who.int/csr/don/31-december-2020-sars-cov2-variants/en [accessed 31.05.2021].


### 2.2. Main characteristics of B.1.351 lineage

B.1.351 lineage was firstly determined in South Africa in early August 2020 and then spread rapidly, too. It is also described as a variant of concern which is may be associated with enhanced transmissibility (Tegally et al., 2020). The genome of this lineage contains several mutations nine of which are located in S protein gene. Among them, K417N, E484K, and N501Y occur in RBD of S protein (Wang et al., 2021). N501Y enhances ACE2 binding affinity as described above. In contrast, K417N and E484K decrease the ACE2-binding affinity by breaking two interfacial salt bridges which ease RBD binding to ACE2 (Cheng et al., 2021). The effect of the E484K substitution is not exactly understood; however, this mutation was associated with escaping neutralizing antibodies (Francisco et al., 2021; Xie et al., 2021). 

B.1.351 lineage also contains D614G mutation in S protein gene which refers to an increased binding affinity for ACE2 that results in enhanced activity of viral entry (Ozono et al., 2021). There are some reports investigating whether a variant including D614G mutation may increase infectivity or transmission of virus or not (Korber et al., 2020; Engelman and Engelman, 2021; Plante et al., 2021; Zhou et al., 2021).

### 2.3. Main characteristics of P.1 lineage (alias of B.1.1.28.1)

First reported in Japan and previously known as B.1.1.28.1, the P1 strain was identified in Brazil as of December 2020. Preliminary data showed the presence of 17 amino acid changes (including 10 in the spike protein), 3 deletions, 4 synonym mutations, and 4 nucleotide insertions in the SARS-CoV-2 P1 strain (Faria et al., 2021). The P.1 strain contains three important mutations in the spike protein receptor binding site, namely K417T, E484K, and N501Y as described above (Dejnirattisai et al., 2021).

It has been reported to have reduced neutralizing activity against the E484K mutation found in P.1 in persons previously infected or vaccinated with other strains of SARS-CoV-2 (Jangra et al., 2021). The first case of reinfection with strain P.1 (containing mutations S: E484K, S: N501Y, and S: K417T) was reported unidentified in a young immunocompromised woman who was infected with strain B.1 virus 9 months ago. It has been reported that nasopharyngeal and pharyngeal samples taken during reinfection of the patient had higher viral loads (SARS-CoV-2 RT-PCR Ct value) than those taken during the first infection, but the patient had equal and moderate symptomatic infections during both attacks.^2^https://virological.org/t/sars-cov-2-reinfection-by-the-new-variant-of-concern-voc-p-1-in-amazonas-brazil/596 [accessed 20 Jan 2021].


Hoffman et al. (2021) reported that mutations in the S proteins of B.1.1.7, B.1.351, and P.1 variants were consistent with strong entry into human cells (Hoffmann et al., 2021). It is of great importance that certain mutations in the P.1 variant may reduce the ability of vaccine-derived antibodies or antibodies formed due to previous natural infection to recognize and neutralize the virus, and thus the ability of this variant to reinfect individuals (Charkiewicz et al., 2021). More epidemic research data is needed to better understand the contagiousness of these strains.

### 2.4. Main characteristics of B.1.617 lineage

B.1.617 variant, also called the “Indian variant”^3^
https://www.sciencemediacentre.org/expert-reaction-to-cases-of-variant-b-1-617-the-indian-variant-being-investigated-in-the-uk/ [accessed 31 May 2021], was firstly determined in India, on 5 October 2020. B.1.617 lineage was described as a VOC in May 2021. B.1.617.1, B.1.617.2, and B.1.617.3 are sublineages of this lineage which are different with their mutations and distribution (World Health Organization, 2021c). Mutations found in this variant are in groups L452R, D614G, P681R, ± (E484Q, Q107H, T19R, del157/158, T478K, D950N) characteristic mutations on the spike protein (World Health Organization, 2021b). This lineage was first reported as a “double mutant” due to the presence of two distinct and significant RBD mutations (E484Q and L452R). E484Q and L452R mutations together cause an escape from the immune mechanism. The appearance of the E484K mutation in this variant has caused immune evasion concerns. Many of these variants are also temporarily linked to increases in cases (Boehm et al., 2021).

## 3. Neurological effects of COVID-19

Neurological symptoms due to COVID-19 disease have been reported in more than 50% of patients. More than 67,529 patients have been researched in 34 different countries, including China, France, Italy, South Korea, Spain, Sweden, the UK, and the United States (US) to date, on the neurological effects of COVID-19 (Wildwing and Holt, 2021). The most common neurological symptoms are fatigue, dizziness, ataxia, dysphagia, taste and smell dysfunctions, anomia, chronic headache, confusion, facial pain, depression-related psychiatric findings, encephalopathy, nonepileptic and epileptic seizures, and neuronal episodes of transient paralysis (Meppiel et al., 2020; Taher et al., 2021). Additionally, a systematic review also described hemorrhagic strokes and cerebral venous sinus thrombosis, especially in critically ill patients with COVID-19. To date, it has been unclear whether the cerebrovascular manifestations are caused by direct viral action or indirect mediation by inflammatory hyperactivation (Fraiman et al., 2020).

**Figure 2:**
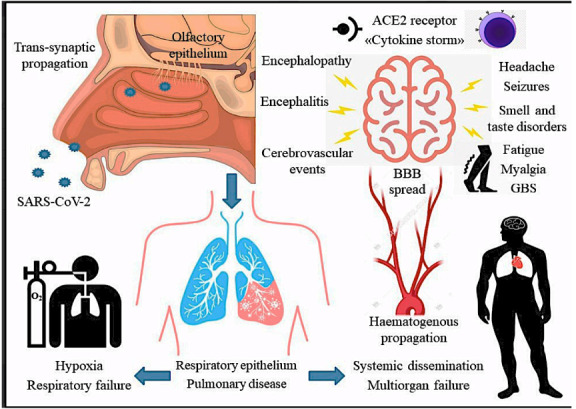
COVID-19, which causes hypoxia by targeting the lung epithelium, can also target olfactory epithelium, create cytokine storm, and cause many neurological symptoms (Pennisi et al., 2020)findings available on its neurological manifestations and their pathogenic mechanisms have not yet been systematically addressed. A literature search on neurological complications reported in patients with COVID-19 until June 2020 produced a total of 23 studies. Overall, these papers report that patients may exhibit a wide range of neurological manifestations, including encephalopathy, encephalitis, seizures, cerebrovascular events, acute polyneuropathy, headache, hypogeusia, and hyposmia, as well as some non-specific symptoms. Whether these features can be an indirect and unspecific consequence of the pulmonary disease or a generalized inflammatory state on the CNS remains to be determined; also, they may rather reflect direct SARS-CoV-2-related neuronal damage. Hematogenous versus transsynaptic propagation, the role of the angiotensin II converting enzyme receptor-2, the spread across the blood-brain barrier, the impact of the hyperimmune response (the so-called “cytokine storm”. Abbreviations: ACE2:angiotensin II converting enzyme receptor-2; BBB: blood–brain barrier; GBS: Guillain-Barré syndrome.

Among the clinical indications for imaging studies for COVID-19, chest computed tomography (CT) and chest radiography have been performed frequently since the first discovery of the disease in Wuhan, China. The cerebrospinal fluid (CSF) analysis, neuroimaging (MRI and CT), and neurophysiological studies [electroencephalogram (EEG), electromyography (EMG) studies, nerve conduction study (NCS)] also reported neurological effects in COVID-19 patients (Yassin et al., 2021). COVID-19 virus penetrates cells with the receptors that are ACE2 and the transmembrane serine protease 2 (TMPRSS2). In our whole body, these receptors are present in the heart, bladder, pancreas, kidney, nose, and in small amounts in the eyes and the brain (Dong et al., 2020). These receptors are particularly abundant in glia, oligodendrocytes, and astrocytes, which are neuronal cells belonging to the cerebral cortex, the striatum, the posterior hypothalamic area, the substantia nigra, and the brain stem (Chen et al., 2021). COVID-19 can pass the blood–brain barrier by transcribal and lymphatic route, axonal and transsynaptic transfer (Figure 2) (Baig et al., 2020; Zubair et al., 2020). Lewis et al. (2020) reported that 6% of 304 patients had the COVID-19 virus in their CSF associated with encephalopathies, quadriplegia due to Guillain–Barré syndrome, multifocal strokes, seizures, headache, vision loss, and neuromuscular disorders. The cytokine storm that occurs in COVID-19 patients damages muscles and the blood–brain barrier and causes encephalopathies headaches; long-term muscular sequelae of COVID-19 include sarcopenia and cachexia, muscle edema and weakness, myositis/rhabdomyolysis, intramuscular hemorrhage, and myalgia (Figure 2) (Paliwal et al., 2020). In addition, autoantibodies against COVID-19 cause damage to endothelial cells, astrocytes, basal ganglia, hippocampus, and olfactory bulbs (Franke et al., 2021). Reichard et al. (2020) found signs of axonal loss, macrophage infiltration, and perivascular encephalomyelitis in the brains of COVID-19 patients. Meppiel et al. reported heterogeneous acute nonvascular lesions in 14 of 192 COVID-19 patients’ MRI findings and found pleocytosis with CSF of 18 patients, 2 of them were encephalitis (Meppiel et al., 2020). Although there are many hypotheses forward like these studies, the neurological complications of COVID-19 are not yet clear according to CSF analysis results, but they may significantly affect the central nervous system. In a recent retrospective cohort examination using electronic health records to compare 6 months of outcomes and the rate of neurological and psychiatric diagnosis (33.62%), including stroke, dementia, Parkinson, neuromuscular or muscle diseases, encephalitis, and Guillain–Barré syndrome, more modest ratios were observed. Moreover, mental disorders were higher in 236,379 COVID-19 patients than in 105,579 patients with influenza and 236,038 patients with other respiratory viruses (Taquet et al., 2021).

Although seizures are a common manifestation of acute severe medical or neurological illness, COVID-19 is associated with seizures. In the metaanalysis study by Kubota et al. (2020), epileptiform discharges were detected in 20.3% of 308 COVID-19 patients. Seizures and status epilepticus were detected at a low rate (2.085% in total). A retrospective multicenter study by Lin et al. (2021) found electrographic seizures in 19 (9.6%) patients, including nonconvulsive status epilepticus (NCSE) in 11, epileptiform abnormalities in 96 (48.7%), clinical seizures during hospitalization in 197 (44.5%) patients with COVID-19. Antony and Haneef published a systematic review about EEG abnormalities were altered mental status (61.7 %), seizure-like events (31.2 %), and postcardiac arrest (3.5 %) from a total of 617 patients (median age was 61.3 years) with COVID-19 (Antony and Haneef, 2020). Lambrecq et al. (2021) reported that 57 patients underwent brain MRI from 78 COVID-19 patients and perfusion abnormalities, acute ischemic lesions, multiple microhemorrhages with white matter-enhancing lesions detected in 41 COVID-19 patients. The authors found that EEG abnormalities were common in the frontal lobe of patients and included focal background slowing, intermittent discharges, and rhythmic delta activity. In conclusion, it is difficult to provide precise data on the short- and long-term neurological effects of the virus due to the small number of research groups performed with EEG and the lack of control groups. In the literature, many studies have detected intact cerebral morphology shown by CT results in COVID-19 patients. Moreover, Galapoulou et al., (2020), Dixon et al., (2020) and De Stefano et al., (2020) reported that acute necrotizing encephalopathy and cerebral microbleeds in CT and MRI results can be reflected in EEG results. In an Italian multicenter retrospective observational study, 34 (31%) patients had acute ischemic infarcts, 6 (6%) had intracranial hemorrhage, 2 had cerebral venous thrombosis, 2 had MS plaque exacerbation, 2 had nonspecific encephalopathy, 2 had Guillain–Barré syndrome, one had Miller Fisher syndrome, and one had acute encephalopathy among 108 COVID-19 patients according to BT and MRI findings (Mahammedi et al., 2020). In another retrospective study with 59 out of 2820 COVID-19 patients reported MRI images, and accordingly, 3 (5.1%) had known white matter lesions from multiple sclerosis, 6 (10.2%) had acute infarcts, 23 (39.0%) had white matter lesions of small vessel ischemic disease, 4 (6.8%) had subacute infarcts, 1 (1.7%) had abnormal basal ganglia signal from hypoxemia, 4 (6.8%) had chronic infarcts, 2 (3.4%) had microhemorrhage in association with chronic infarcts, and 2 (3.4%) had microhemorrhage associated with acute or subacute infarcts (Freeman et al., 2021). The authors also noted that MRI findings were affected by comorbidities of hypertension and type 2 diabetes mellitus in seven COVID-19 patients.

ACE2 and TMPRSS2 receptors are highly present in the olfactory epithelium but absent in olfactory sensory neurons. Sustentacular cells in the smelling epithelium are the main target of COVID-19 (Meunier et al., 2021). Although the WHO reported the loss of smell and taste from neurological findings related to COVID-19 disease on May 4, 2020, whether odor and taste loss can be diagnostic criteria for COVID-19 has been a matter of debate^4^Coronavirus disease (COVID-19) (who.int) [Accessed 7 April 2021].. In a prospective study, 67 patients were reported to experience smell and taste loss from 94 COVID-19 patients, of whom 3 (4.4%) had taste loss, 30 (44.7%) had both smell and taste impairment, and 34 (50.7%) had smell impairment (Salcan et al., 2021). Similarly, an updated review comprising a large-scale study on 40,000 patients from a total of 104 studies reported that anosmia is the most widespread symptom in COVID-19 infection in different societies (Meunier et al., 2021). In a large-scale study, new approaches based on machine learning were applied to the visual analogue scale (VAS) results of 777 COVID-19 patients. The results reached an average of 80% accuracy, 82% sensitivity, and 78% specificity when VAS was used in the diagnosis of COVID-19 disease (Callejon-Leblic et al., 2021). In a recent metaanalysis involving 107 studies, anosmia was observed in 12,038 of 32,142 COVID-19 patients (prevalence of 38.2%), whereas dysgeusia was reported in 11,337 of 30,901 COVID-19 patients all over the world (from 101 studies in total) with the prevalence of 36.6% (Mutiawati et al., 2021). The frequency of anosmia and dysgeusia is quite common in respiratory infections due to COVID-19 compared to others. As a result, current studies are generally concerned about the short-term (<6 months) results; however, future studies are needed to elucidate the long-term (>6 months) neurological symptoms of COVID-19. Consequently, as the disease affects mental health and the brain, increase in need for mental health services and neurological rehabilitation services should be expected.

## 4. Current COVID-19 treatments

### 4.1. Drugs for COVID-19

**Figure 3:**
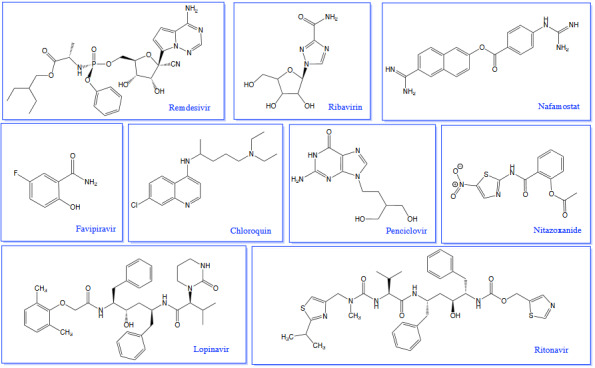
Structures of antiviral drugs prominent in the Covid19 pandemic.

Corona virus has been effective all over the world for about 2 years, and studies on vaccines and drugs are intensely carried out in order to effectively fight the pandemic. Since viruses are constantly mutating in viral infections, the development of antiviral drugs for now and beyond has remained a very important challenge for the scientific world. FDA supported the therapeutic efficiency of ribavirin (RBV), favipiravir (FVP), remdesivir (RDV), penciclovir (PCV), chloroquine, nafamostat, and nitazoxanide against SARS-CoV-2 strain based on in vitro trials. Currently, several drugs such as hydroxychloroquine (HCQ), RBV, FVP, lopinavir/ritonavir (LPV/r), RDV, and oseltamivir have been suggested as effective treatments for SARS-CoV-2 (Figure 3) (Kocayiğit et al., 2021). However, in this coronavirus pandemic, no specific drugs have been approved for COVID-19 yet.

#### 4.1.1. Nucleoside analogs

Nucleoside analogs, RNA-bound RNA polymerase enzyme blocking and including human coronaviruses in a broad spectrum of RNA viruses are adenine or guanine derivatives which affects the structure of viral RNA. RDV, FVP, RBV, penciclovir, nitazoxanide, and nafamostat are among drugs that are being tested in controlled randomized clinical trials for COVID-19. Some of them are detailed below.

RDV is an important nucleotide analog originally developed for the treatment of Ebola virus disease and also it has antiviral activity against multiple phyllo-, paramyxo-, pneumo-, and corona viruses (Sheahan et al., 2017). RDV, which has broad spectrum antiviral activity, is an RNA-dependent RNA polymerase (RdRps) inhibitor, and it reduces viral replication with early termination of RNA transcription and thus improves pulmonary function by reducing viral load in the lungs (Yurdakök Dikmen et al., 2020) and therefore was suggested as a therapeutic agent against SARS-CoV-2. As it was also determined to be a highly effective agent against MERS-CoV by reducing the viral loads in mice after 12 days of administration and therefore supporting regaining the normal pulmonary functions. A combination of RDV and chloroquine in in vitro research demonstrated effective inhibition of SARS-CoV-2 growth in Vero E6 cells. The USA, Norway, and France continue clinical studies to evaluate the efficacy of RDV for COVID-19, it has been used to treat COVID-19 patients in the USA and Singapore. In the USA, RDV was given intravenously and first subjects of COVID-19 recovered (Gordon et al., 2020; Holshue et al., 2020). Drug-related randomized controlled trials have been continued. In in vitro VERO cell culture studies, a combination of RDV and chloroquine have been shown to be effective against SARS-CoV-2 (Deshpande and Mali, 2020). A metaanalysis study reported that RDV may have good impact on clinical improvement in hospitalized COVID-19 patients (Jiang et al., 2021). The WHO declared that RDV has little effect on mortality, mechanical ventilation, viral clearance, time to clinical improvement, and duration of hospitalization due to COVID-19.^5^World Health Organization (2021). Therapeutics and COVID-19: living guideline, 31 March 2021 [online]. Website https://apps.who.int/iris/handle/10665/340374 [accessed 08.04.2021].


FPV is a heteroaromatic molecule with antiviral activity carrying carboxy amide, fluorine, and hydroxy groups and shows fluorescent properties. It is a purine analog and pyrazine carboxamide derivative antiviral agent. Originally developed to treat influenza, FPV targets RdRps and blocks the replication of rhinoviruses. In addition to influenza, recent studies in the literature report that FPV acts as a broad-spectrum medication that prevents the multiplication of flavivirus, rhinovirus, poliovirus, arena viruses, and filovirus. FPV shows its effect by selectively and strongly inhibiting RdRp in RNA viruses. Wang et al. (2020) reported that it has been used as an effective treatment for COVID-19 patients in their observational study. In addition to FPV, penciclovir and RBV are effective in reducing SARS-CoV-2 infection in vitro. In February 2020, a report by Cai et al. (2020) showed a significantly faster mean time to viral clearance by FVP compared to LPV/r (4 days vs 11 days, p < 0.001). Similarly, Kocayiğit et al. observed that the FVP group had a shorter duration of hospitalization than the LPV/r group (Kocayiğit et al., 2021). Favipiravir should be used in early stage with quick diagnosis in selected patients (Deshpande and Mali, 2020). 

Another antiviral drug RBV is a synthetic purine nucleoside analogue having an antiviral activity against SARS-CoV-2 and against a broad-spectrum of both DNA and RNA viruses (Graci and Cameron, 2006; Deshpande and Mali, 2020). RBV can potentially act on numerous steps of the virus life cycle: inhibition of translation due to reduction in cellular GTP pools or incorporation as a cap analogue which inhibits translation; inhibition of genome or transcript capping, by suppression of GTP synthesis or direct competition; inhibition of RNA synthesis directly via active-site binding or reduction of GTP synthesis; ambiguous incorporation into RNA causing increased mutation and production of nonviable genomes; or enhancement of the antiviral immune response, preventing spread and pathogenesis (Graci and Cameron, 2006). The effect of RBV against SARSCoV-2 is being investigated in a clinical trial in Hong Kong. It degrades viral RNA by inhibiting inosine monophosphate dehydrogenase to reduce the production of guanosine (Deshpande and Mali, 2020). 

PCV, an acyclic guanosine analogue, was developed for the treatment of various herpesvirus infections in Beecham Pharmaceuticals Laboratories. Penciclovir-triphosphate is PCV’s active metabolite and inhibits the replicative function of the viral DNA polymerase competitively. Unlike acyclovir, penciclovir is not considered a DNA chain terminator due to the presence of the 3′-OH group in its structure. Penciclovir has range spectrum antiviral activity against VZV, HSV, and EBV (Pastuch-Gawołek et al., 2019). Yin et al. also reported that FPV, RBV, and penciclovir are inhibitors of the COVID-19 RdRp, an essential enzyme for the viral replication (Yin et al., 2020).

Nitazoxanide (NTZ) is a range spectrum antiparasitic and antiviral medicine used for the treatment of various protozoal, helminthic, and viral infections (White Jr., 2004; Rossignol, 2014). NTZ is a thiazolide derivative with antiinfective effect and it inhibits replication of influenza viruses, including neuraminidase inhibitor-resistant strains, blocking the maturation of viral hemagglutin at the posttranslational level. In cell culture research, NTZ acts synergistically with neuraminidase inhibitors (Haffizulla et al., 2014). Therefore, new protocols and therapeutic approaches with combinations of nitazoxanide/oseltamivir (Rossignol, 2014), nitazoxanide/hydroxychloroquine (Haffizulla et al., 2014), and nitazoxanide/azithromycin (Kelleni, 2020) have been clinically tested against COVID-19.

Hydroxychloroquine (HCQ) is classified within the 4-aminoquinoline drug families. Chloroquine, an antimalarial drug, has been determined to be beneficial against COVID-19. It was used for the treatment of COVID-19 patients. It prevents the entry of the virus by changing the structural configuration of cell receptors and binding to the cellular receptors competitively. It can alter the glycosylation of ACE2 cellular receptors required for SARS-CoV-2 entry into the cell. In other words, this drug can also reduce the synthesis of sialic acid receptors to prevent SARS-CoV-2 from binding to host cells (Frediansyah et al., 2021). However, the WHO reported that hydroxychloroquine did not reduce duration of mechanical ventilation and mortality. In addition, taking hydroxychloroquine to treat COVID-19 may increase the risk of heart rhythm problems, blood and lymph disorders, liver problems, and kidney injury^6^https://clinicaltrials.gov/ [accessed 10.04.2021].

In addition to the drugs mentioned above, the FDA has approved the transcription inhibitors (NtRTIs) such as tenofovir alafenamide, tenofovir, disoproxil, adefovir, abacavir, didanosine, and ganciclovir. These antiviral drugs with similar structural properties with RDV and/or RBV may make them possible antiviral drugs against COVID-19. Additionally, some other transcriptase inhibitors like NRTIs (lamivudine, zidovudine, stavudine, zalcitabine, emtricitabine, and azvudine) and NNRTIs (delavirdine, rilpivirine, nevirapine, and efavirenz) have been approved by the FDA (Lamprou, 2020).

Currently, 5316 randomized controlled clinical trials are being conducted for COVID-196. In the randomized controlled clinic studies for COVID-19, there are no specific antiviral agents proven across the board reliability and efficacy yet. However, in order to develop an effective treatment model, the effects of existing antiviral drugs on COVID-19 are being investigated. In the global pandemic process, antivirals such as RDV, FPV, HCQ, and RBV have attracted more attention in the treatment of COVID-19 patients. Especially, RBV has a versatile antiviral effect. RDV has been approved by FDA for treating COVID-19. FPV has been approved in China, Russia, and India for COVID-19, and it is known that the drug reduces the treatment time from 11 days to 4 days (Cai et al., 2020; Yin et al., 2020).

Antiviral drugs applied in a short time following the onset of symptoms can reduce viral load and contagiousness, especially if drugs that have proven to have antiviral effects, such as RBV that has a versatile antiviral effect, FPV that was developed to treat influenza, and RDV that was developed for the treatment of Ebola virus, are used at an early stage. It has been reported in clinical trials that they provide an effective treatment. However, antiviral drugs known in clinical trials are not at the desired level on mortality, mechanical ventilation, viral clearance, and duration of hospitalization due to COVID-19. 

Although the past strategy in combating antimicrobial pathogens is particularly on target pathogen genes and proteins, viral genes are exposed to rapid mutation and show resistance to drugs due to their intrinsic nature. When antiviral drugs are not administered in the appropriate replication period, they cause failure and often resistance development; therefore, drugs affecting the host become more prominent in the clinic trials for COVID-19. Especially in the selection of antiviral drugs, there may be several benefits of targeting host factors such as much lower DNA polymerase mutation rates than those of RNA and the ability to control cytokine storm and have a wider antivirus spectrum of targeted host factors. Therefore, targeting host factors can be a plausible approach for developing COVID-19 therapeutics or other emerging viruses in the future (Cai et al., 2020).

Consequently, treatment protocols in the world are constantly updated with the results of controlled randomized clinical trials. Individualized medicine is an important treatment approach since there is no uniform drug application in treatment due to individual differences such as changing phenotype, other chronic diseases and also in advanced stages of the disease, combinations of viral drugs are tried to create synergistic effect.

### 4.2. Currently used COVID-19 vaccines and their efficiency 

There have been more than 200 COVID-19 vaccine candidates under development since December 2020. A minimum of 52 candidate vaccines are in the human trial phase and 18 vaccines are under efficiency trials. When developing a vaccine for COVID-19, three main approaches were taken: the whole virus, parts of the virus targeting the immune system, and the viral gene. In summary, the vaccine types currently being studied on COVID-19 are inactivated, live-attenuated, viral vector, subunit, and nucleic acid vaccines. Some COVID-19 vaccines have been developed using an approach that is not live virus vaccines. The material contains messenger RNA (mRNA) and viral proteins that do not interfere with human DNA^7^The World Health Organization (2021). Safety of COVID-19 Vaccines [online]. Website https://www.who.int/news-room/feature-stories/detail/safety-of-covid-19-vaccines [Accessed 7 April 2021].
.

**Figure 4:**
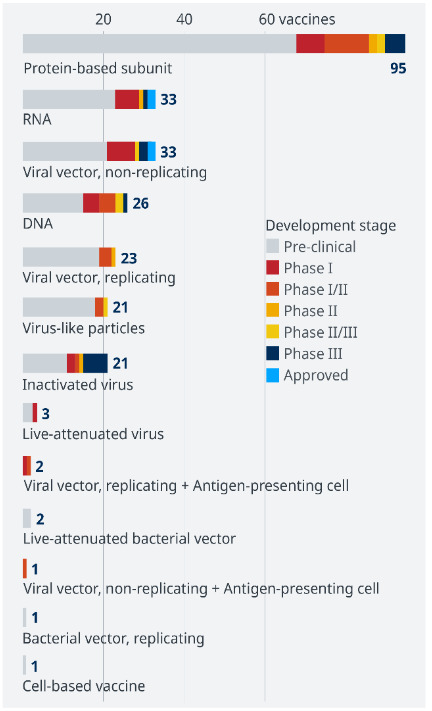
Current status of COVID-19 vaccine trails.

Currently, there are five FDA- and EMA-approved vaccines (Figure 4); AstraZeneca (ChAdOx1 nCoV-19) and Gamaleya developed adenovirus-expressing spike vaccine; Pfizer/BioNTech (bnt162b2 mRNA vaccine) and Moderna (mRNA-1273 vaccine) developed RNA vaccines expressing the spike glycoprotein; Sinopharm and Sinovac developed an inactivated virus vaccine with alum as an adjuvant (Kim et al., 2021). Pfizer/BioNTech, Gamaleya, Moderna, Sinopharm, and AstraZeneca have respectively reported 95%, 92%, 94.5%, 79%, and 70% vaccine efficacy (Polack et al., 2020). Sinovac (owned by the Chinese company) announced vaccine efficacies ranging from 50% to 91% (for the same product) for many countries participating in the efficacy trials, but the full data has not yet been published as it is still under peer review (Voysey et al., 2021)^8^https://investor.lilly.com/news-releases/news-release-details/lillys-neutralizing-antibody-bamlanivimab-ly-cov555-receives-fda [accessed 31.05.2021]. Although the safety studies of vaccines will continue until the end of the application all over the world, the main question currently in mind is whether these vaccines will provide the same reliability in patients with chronic diseases or under various treatments. Waissengrin et al. (2021) reported that the Pfizer/BioNTech vaccine was offered to 170 patients (cancer and different comorbidities) treated with immune checkpoint inhibitors. Of these, 137 (81%) patients received the first dose of vaccine, of whom 134 (98%) received the second dose. Three people died after the first dose. A very large-scale clinical trial showed that COVID-19 vaccine Moderna was 90.9% effective in participants at risk of severe illness, including those with heart disease, chronic lung disease, obesity, diabetes, liver disease, or HIV infection. High efficacy in this vaccine was also maintained across racial, gender, and ethnic groups (Baden et al., 2021). However, chronic and autoimmune diseases can also be triggered by vaccines. Immune mechanisms such as antigen presentation, antiidiotypic status, cytokine production, polyclonal activation of B cells, and epitope dissemination play a role in both autoreactivity and antiinfectious immune response. Individuals under immunosuppressive therapy or those diagnosed with an immunocompromising condition were generally excluded in approved COVID-19 vaccine trials (Polack et al., 2020; Logunov et al., 2021). There are not enough reports to discuss the efficiency of approved vaccination on chronic, other infectious and autoimmune diseases. There are some important questions to ask regarding individuals with such diseases:

Could vaccines have serious side effects on these patients?

Will vaccines be harmful or beneficial, especially in combination with immunosuppressive, chemo, and other therapies?

Does the vaccine trigger diseases in those with low immunity and latent autoimmune condition?

Careful attention should be paid to vaccines in individuals with chronic diseases and pharmaco-epidemiological studies are needed for each vaccine delivery system urgently. Especially special prospective COVID-19 vaccine trials involving patients with advanced stage kidney, liver, biliary, intestinal, and comorbid disease, and their transplant recipients are urgently needed and likely to emerge soon.

### 4.3. Neutralizing antibody therapy 

The degree and duration of the protection provided by the immune response that develops in those exposed to SARS-CoV-2 or those who have had an infection is still one of the controversial issues. One of the most important branches of the immune response in this type of infection is the development of antibodies that recognize the virus. Antibodies that block binding to the Angiotensin Converting Enzyme-2 (ACE-2) receptor in the host cell membrane are antibodies that recognize and bind to the spike protein of the virus (Oral et al., 2002; Gattinger et al., 2021). Monoclonal antibodies come first among neutralizing antibodies. Other important mechanisms in neutralizing the virus’s binding ability to the receptors such as transmembrane protease serine 2 (TMPRSS2), CD147, and CD26, and to directly kill the virus in cultures or in vivo models (Radzikowska et al., 2020; Gattinger et al., 2021). 

The high-tech approach is to manipulate antibody-producing B cells obtained from the blood of people with COVID-19. The most important of these are monoclonal antibodies (MoAb) (Hurt and Wheatley, 2021). The production of MoAb is based on the principle that each B cell produces a unique antibody. The clone of a B cell that produces quality antibodies (for example, which binds to the antigen with high affinity) can be expanded and used by producing antibodies specific to a single antigenic determinant (epitope) called MoAb. These antibodies can be cloned from the B cell repertoires of healed patients or can be produced in vitro by genetic engineering (Beigel, 2018). MoAbs against viruses can be classified in two main groups according to their targeted location: Those targeting the receptor structure, or inhibit virus binding or entry of the host virus; antibiotics with virus replication and transcription (Owji et al., 2020).

Due to the homology between SARS-CoV and SARS-CoV-2, it was initially expected that the SARS-CoV antibodies would show SARS-CoV-2 cross-reactivity. However, there are still contradictions about this because unlike SARS-CoV, there are some highly protected regions in SARS-CoV-2. For example, the C-terminal part of the SARS-CoV-2 RBD region is quite different from that of SARS-CoV. In addition, S1 and S2 subunits not found in SARS-CoV, in SARS-CoV-2, these units are the furin cleavage site. These differences do not affect the ability of the virus to interact with the ACE2 receptor, although there are differences in the ability of the antibodies developed to neutralize the virus (Tian et al., 2020; Yuan et al., 2020). As a matter of fact, the computational-based analysis of the epitopes of the two viruses showed that 85.3% of the epitopes belonging to the SARS-CoV-2 spike were different from those of SARS-CoV (Zheng and Song, 2020). To date, studies have shown that only a few (F6G19, CR3022, and 47D11) of about 25 different monoclonal antibodies developed against SARS-CoV can neutralize both SARS-CoV and SARS-CoV-2 (Owji et al., 2020). For this reason, research on neutralizing antibodies specific to SARS-CoV-2 is also ongoing. In one of these, it has been shown that the developed SARS-CoV-2 RBD-specific monoclonal antibodies have a very strong neutralizing property (Ju et al., 2020). However, it has been demonstrated that these antibodies do not show cross-reactivity with SARS-CoV and MERS-CoV. Again, it has been shown that the use of cocktails of MoAbs that have the ability to specifically bind to different epitopes in RBD can be more effective in neutralizing SARS-CoV-2 as well as providing protection against escape mutants (Wu et al., 2020). Antibodies also can destroy infected cells, which can change the structure of a protein or through the effector functions required for viral entry. Various antibodies against the spike protein have been developed to block the binding of the receptor to the host cell membrane^8^ (Wong, 2020). Anti-SARS-CoV-2 antibodies will bind virus and free from infection with the hope of effectively detecting a force sufficient to inactivate were isolated from large patient population (Twomey et al., 2020). Antibody cocktails, expanding the potential neutralization, can provide protection against future mutations in the virus’s spike protein can target different epitopes. These cocktails were used for pneumonia, and they showed an activity to decrease the viral load in in vitro as well as in animal models of injury (Hansen et al., 2020; Regeneron, 2021).

MoAb on cells and animals in preclinical studies in early 2020 shows promise against COVID-19. Last September, the first results of these studies came out and several monoclonal antibodies have since received emergency use from the FDA (U.S. Food and Drug Administration, 2020).

Various antibody products have been developed to treat problems caused by cytokine storm and COVID-19 pneumonias. These treatments, IL-6 (Levilimab) (NCT04397562), TLR4 (EB05) (NCT04401475), CXCL10 (EB06) and angiopoietin-2 (Ang2) (LY3127804) (NCT04342897) comprise targeting. Type-I and type-III interferon or IL-6R targeting TLR4 controls and suppresses the proinflammatory response in cytokine storm in patients (Sallenave and Guillot, 2020).

According to published studies evaluating the efficacy and safety of the use of interferon β-1a (IFN β-1a) in the treatment of COVID-19, interferon β-1a at a dose of 44 µg was administered subcutaneously to patients in certain sequential processes. Significantly, it reduced mortality and hospital stay in early application. The use of interferon β-1a in combination in the treatment of COVID-19 should be supported (Dastan et al., 2020; Davoudi-Monfared et al., 2020).

In addition, the bradykinin storm is a subspecies of the kallikrein-kinin system. It multiplies in the plasma as a result of the division of cells produced in the liver by means of proteolytic enzymes. ACE has a higher affinity for bradykinin than angiotensin I. In cases where ACE is low, bradykinin is preserved. Due to decreased ACE gene expression in COVID-19 patients, there will be increases in bradykinin receptors and enzymes (Van de Veerdonk et al., 2020). Bradykinin receptor antagonists are of potential interest, as bradykinin suppresses interferon production, thereby increasing cellular inflammation. Therapeutics trying to correct the imbalance in the pathway of angiotensin and kallikrein products are promising areas of research in COVID-19 (Roche and Roche, 2020).

First of all, these antibodies are thought to be more effective than many other drugs because they originate from the blood of people who have had COVID-19 and are obtained by selecting the most effective ones. They can be produced very quickly compared to other drugs and vaccines. As soon as they are administered, they can provide effective protection against infection, but their effectiveness decreases over weeks or months due to the certain half-life of antibodies. There are no big differences in effect in children and the elderly. In addition, they are effective in people with weak immune systems where some vaccines cannot be administered or an effective immune response cannot be established with vaccines. Production of monoclonal antibodies is more complex and expensive than other types of drugs. Therefore, they are the most expensive and hardest-to-reach drugs in the world.

A disadvantage of the MOAB is that they can lose their impressive efficacy against new variants. Mutated variants escape antibodies produced in those previously infected with COVID-19. Researchers are developing cocktails of monoclonal antibodies to overcome the resistance of these new variants.

## 5. Conclusion

In conclusion, the COVID-19 epidemic has been on the agenda of the world since the day it caused a global health problem. Scientists and health organizations are making great efforts to control the epidemic and take therapeutic/preventive measures. In this study, we presented up-to-date information, especially in terms of variants, neurological symptoms, drugs, vaccines, and neutralizing antibodies. Treatment and prophylaxis studies continue rapidly all over the world.
